# ADCK1 is a potential therapeutic target of osteosarcoma

**DOI:** 10.1038/s41419-022-05401-8

**Published:** 2022-11-12

**Authors:** Bao-biao Zhuo, Lun-qing Zhu, Chen Yao, Xi-hua Wang, Shi-xian Li, Rong Wang, Yuan Li, Zhuo-yan Ling

**Affiliations:** 1 Department of Pediatric Surgery, Affiliated Hospital of Xuzhou Medical University, Department of orthopedics, Xuzhou Children’s Hospital, Xuzhou, China; 2 Department of Orthopaedics, Children’s Hospital of Soochow University, Suzhou, China; 3 Department of Orthopedics, Affiliated Hospital of Nanjing University of TCM, Jiangsu Province Hospital of TCM, Nanjing, China; 4 Xuzhou Medical University, Xuzhou, China; 5 Department of Ultrasound, Affiliated Hospital of Xuzhou Medical University, Xuzhou, China; 6 Department of Orthopedics, the Second Affiliated Hospital of Soochow University, Suzhou, China

**Keywords:** Targeted therapies, Sarcoma

## Abstract

We here showed that ADCK1 (AarF domain-containing kinase 1), a mitochondrial protein, is upregulated in human osteosarcoma (OS) tissues and OS cells. In primary and established OS cells, ADCK1 shRNA or CRISPR/Cas9-induced ADCK1 knockout (KO) remarkably inhibited cell viability, proliferation and migration, and provoked apoptosis activation. Conversely, ectopic ADCK1 overexpression exerted pro-cancerous activity by promoting OS cell proliferation and migration. ADCK1 depletion disrupted mitochondrial functions in OS cells and induced mitochondrial membrane potential reduction, ATP depletion, reactive oxygen species production. Significantly, ADCK1 silencing augmented doxorubicin-induced apoptosis in primary OS cells. mTOR activation is important for ADCK1 expression in OS cells. The mTOR inhibitors, rapamycin and AZD2014, as well as mTOR shRNA, potently decreased ADCK1 expression in primary OS cells. In nude mice, the growth of subcutaneous pOS-1 xenografts was largely inhibited when bearing ADCK1 shRNA or ADCK1 KO construct. Moreover, ADCK1 KO largely inhibited pOS-1 xenograft in situ growth in proximal tibia of nude mice. ADCK1 depletion, apoptosis activation and ATP reduction were detected in pOS-1 xenografts bearing ADCK1 shRNA or ADCK1 KO construct. Together, the mitochondrial protein ADCK1 is required for OS cell growth and is a novel therapeutic target of OS.

## Introduction

Osteosarcoma (OS) is a bone malignancy [1, 2]. The current clinical treatments of OS include neoadjuvant chemotherapy, radiotherapy and surgery [3, 4]. Due to the use of chemotherapy drugs, including doxorubicin, cisplatin and methotrexate [3–5], the prognosis of the OS patients has been significantly improved [6]. However, the five-year overall survival has remained largely unchanged in the past three to four decades [6]. For the OS patients with local recurrence and metastasis, the 5-year-survival rate is only about 20% [2, 7]. In order to develop novel therapeutics to improve patients’ prognosis [8, 9], it is extremely important to further explore the key genes for the progression and tumorigenesis of OS [10–12].

ADCK1 (AarF domain-containing kinase 1) belongs to the mitochondrial ADCK family kinases and localizes at the inner membrane of mitochondria [13]. In *Drosophila* ADCK1 was reported to utilize YME1-like 1 ATPase (YME1L1) to control optic atrophy 1 (OPA1) and inner membrane mitochondrial protein (IMMT) in maintaining mitochondrial functions and structure of muscles [14]. Conversely, ADCK1 knockdown or loss-of-function mutations induced mitochondrial functional abnormalities, leading to mitochondrial membrane potential reduction, ATP depletion, reactive oxygen species (ROS) production and oxidative stress in *Drosophila*, as well as mitochondria structural abnormality and apoptosis [14]. *ADCK1* overexpression was able to restore the membrane lipid levels and growth defects in *Mdm10* mutants in *Drosophila* [15]. ADCK1 in the trachea is necessary and sufficient for the survival of larva *Drosophila*. *ADCK1* mutants caused the larval lethality and double mouth hooks, possibly due to the decreased levels of the steroid hormone ecdysone [13]. These studies of ADCK1 provided implications of the potential physiological functions of this mitochondrial protein in maintaining mitochondrial structures and functions as well as in promoting development and viability in *Drosophila*.

It is not clear whether ADCK1 also affects the functions of mitochondria and behaviors of human cells, and whether its expression is altered in human cancer cells. A very recent study by Ji et al., reported that ADCK1 is overexpressed in clinical colon cancer specimens and involved in colon cancer tumorigenesis [16]. ADCK1 silencing, by the shRNA strategy, potently inhibited colon cancer cell colony formation and infiltration as well as the in vivo tumorigenesis, migration, and organoid formation [16]. ADCK1 directly associated with TCF4 (T-cell factor 4) to activate the β-catenin/TCF signaling pathway, thus promoting expression of β-catenin downstream genes [16]. Its expression and potential functions in OS are examined in the present study.

## Materials and methods

### Reagents

Cell Counting Kit-8 (CCK-8) was from Dojindo Co. (Kumamoto, Japan). Puromycin, hydrogen peroxide (H_2_O_2_), antibiotics, medium, serum, doxorubicin, cisplatin, N-Acetyl-L-cysteine (NAC), ATP, polybrene as well as rapamycin, AZD2014 and the caspase inhibitor (z-VAD-fmk) were provided by Sigma-Aldrich Chemicals (St. Louis, Mo, USA). EdU (5-Ethynyl-2’-deoxyuridine), DAPI (4’,6-diamidino-2-phenylindole), TUNEL (Terminal deoxynucleotidyl transferase dUTP nick end labeling), JC-1 and CellROX fluorescence dyes were provided by Invitrogen Thermo-Fisher (Shanghai, China). Antibodies for cleaved caspase-3 (#9664), cleaved-poly (ADP-ribose) polymerase 1 (PARP1) (#5625) and β-actin (#3700) were from Cell Signaling Tech China (Danvers, MA, USA). Antibodies for phosphorylated (“p”) p70 S6 kinase 1 (S6K1, Thr 398) (9209), S6K1 (9202), p-S6 ribosomal protein (S6, Ser 235/236) (2211), DNA Damage Antibody Sampler Kit (#9947), S6 (2317) and mTOR (2972) were all also from Cell Signaling Tech (Danvers, MA, USA). ADCK1 (TA308421) and ADCK2 (TA343272) antibodies were provided by OriGene (Wuxi, China). The anti-ADCK3 antibody was obtained from Abcam (ab230897, Shanghai, China). All mRNA primers were provided by Genechem (Shanghai, China).

### Cell culture

The established OS cell lines (U2OS and MG63), the primary human OS cells derived from three patients, pOS-1, pOS-2 and pOS-3, as well as the primary human osteoblasts were provided by Dr. Cao at Soochow University [17, 18], and cells were cultured using the described protocols [17, 18]. Using primary human cells was approved by Ethics committee of Xuzhou Medical University.

### Human tissues

Sixteen written-informed consent primary OS patients with tumor resection surgery, administrated at authors institutions, were enrolled in this study. OS tumor tissues and the matched adjacent normal bone tissues were obtained at the time of surgery. Tissues were incubated with the described lysis buffer [19] and stored in liquid nitrogen. The human or xenograft tissue slides (4 µm in thickness) were incubated with anti-ADCK1 antibody overnight at 4 °C and the green fluorescence secondary antibody 37 °C for 1 h. Alternatively, the mitochondria were stained with MitoTracker Red (Invitrogen,) and cell nuclei were stained with DAPI (green fluorescence dye). The tissue fluorescence slides were tested by a confocal fluorescence microscope (Zeiss). Protocols of using human tissues were approved by the Ethic Committee of Xuzhou Medical University, in according to the principles of Declaration of Helsinki.

### shRNA

The lentiviral particles encoding the ADCK1 shRNA (sequence listed in [16]), the ADCK2 shRNA, the ADCK3 shRNA, or the mTOR shRNA were obtained from GeneChem Inc. (Shanghai, China). According to the instruction, OS were plated into a six-well plate at 8.5 × 10^4^ cells per well for 24 h. Cells were then infected with the virus at the multiplicity of infection (MOI) of 20. After 24 h, the transfection mixture was replaced with normal complete growth medium. To establish stable cells, the lentivirus-infected cells were selected by puromycin (2 μg/mL) for 96 h. The expression of ADCK1 or mTOR in the stable cells was verified by qRT-PCR and Western blotting assays.

### ADCK1 knockout

OS cells were transfected with Cas9-expressing construct (GeneChem, Shanghai, China), and stable cells established after puromycin selection. The small guide RNA (sgRNA) targeting ADCK1 (sg-1, targeted DNA sequence, AAGTACTCCTCTGAGCCATA, PAM sequence, AGG. sg-2, targeted DNA sequence, TCTGGACTACCTGTTGCCAG, PAM sequence, AGG) was inserted into the lenti-CRISPR/Cas9-puro plasmid [20]. The CRISPR-ADCK1-KO construct, the psPAX2 packaging plasmid, and the pMD2.G envelope plasmid were mixed in a 2:1:1 ratio diluted in serum-free medium together with Lipofectamine 3000, and were transfected into HEK293T cells. The virus-containing medium was then collected after 24 h and virus was filtered and was utilized to infect Cas9-expressing cancer cells. The infected cells were then distributed into 96-well plates. Single stable ADCK1 KO cells were then established. ADCK1 *mRNA* and protein expression in the stable cells was always tested by qRT-PCR and Western blotting assays.

### ADCK1 overexpression

The lentiviral particles encoding the wild-type *ADCK1* cDNA or the ADCK1 shRNA-resistant *ADCK1* cDNA (containing mutations at the shRNA-targeting sites) were constructed by Hanbio Co. LTD (Shanghai, China). OS cells were seeded into 12-well plates (in polybrene-containing complete medium) and infected with the lentivirus at MOI of 30. After 48 h, the medium was replaced with complete growth medium. To establish stable cells, the lentivirus-infected cells were selected by puromycin (2 μg/mL) for 96 h. ADCK1 overexpression in the stable cells was verified by qRT-PCR and Western blotting assays.

### The kinase-dead ADCK1 (“ADCK1-KD”)

Site-directed mutagenesis of ADCK1 (pA164G, K183I, D315A) was carried out through a Fast Mutagenesis System Kit (Cat# FM111-01, TransGen Biotech, Beijing). The mutations were confirmed by DNA sequencing. The sequence was cloned into pcDNA3.1(+). The mutant ADCK1 construct, the psPAX2 packaging plasmid, and the pMD2.G envelope plasmid were mixed in a 2:1:1 ratio diluted in serum-free medium together with Lipofectamine 3000, and were transfected into HEK293T cells. The generated lentivirus was then added to the cancer cells and stable cells were formed after puromycin selection.

Other assays, including quantitative real-time PCR (qRT-PCR), Western blotting including CCK-8 viability, trypan blue staining of cell death, colony formation, nuclear EdU staining, “Transwell” assays, TUNEL staining, Annexin V FACS, caspase-3 activity assay, single strand DNA (ssDNA) ELISA, Histone DNA ELISA and JC-1 staining were described in detail in our previous studies [17, 18]. The uncropped blotting images of this study were listed in Fig. S3.

### ROS and lipid peroxidation assays

OS cells, with or without applied genetic modifications, were placed onto 12-well plates at 0.5 × 10^5^ cells per well and cultivated for indicated time periods. Cells were then washed once with PBS and stained with the applied fluorescence dyes (DCF-DA or CellROX, dissolved in the medium). After extensive washes, fluorescence images were visualized under a fluorescence microscopy (Leica), and its intensity was detected by a fluorescence spectrophotometer (F-7000, Hitachi-Hightech). A thiobarbituric acid reactive substances (TBAR) activity assay kit was employed to examine cellular lipid peroxidation levels using the protocol described [21, 22].

### ATP contents

OS cells were plated into 12-well plates at 0.5 × 10^5^ cells per well for 72 h. An ATP assay kit (Biyuntian, Wuxi, China) was utilized to quantify ATP levels according to the attached protocols.

### Constitutively active mutant Akt1

A recombinant adenoviral construct encoding the constitutively active Akt1 (caAkt1, S473D) was from Dr. Xu’s group [23], transduced to pOS-1 cells. Puromycin was added to select stable cells, where caAkt1 expression was verified by Western blotting assays.

### Xenograft studies

The 5–6 week old BALB/c-nude mice (half male half female, 18.2–19.2 g weight) were provided by the Experimental Animal Center of Xuzhou Medical University (Yangzhou, China). The pOS-1 cells with applied genetic modifications (at 6 × 10^6^ cells per mouse, in Matrigel-containing basic medium) were subcutaneously (*s.c*.) injected to the flanks of nude mice. The xenograft tumors were established within two weeks. Thereafter, tumor volumes and mice body weights were recorded every six days. The detailed protocols for the immunohistochemistry (IHC) assay in the xenograft slides were reported early [24]. For in situ OS model, the pOS-1 cells (at 3 × 10^6^ cells per mouse) were injected to the proximal tibia of the nude mice [17]. After 30 days, in situ tumors were visualized under X-ray film. The animal protocols were approved by Institutional Animal Care and Use Committee (IACUC) and Ethics Committee of Xuzhou Medical University.

### Statistical analyses

All data were with normal distribution and were presented as mean ± SD (standard deviation). Two-sided student’s *t*-tests (for comparison of two groups, Excel 2007) or analysis of variance (ANOVA) tests (for comparison of multiple groups, SPSS23.0) were utilized to assess statistically significant differences. Values of *P* < 0.05 were considered as statistically significant.

## Results

### ADCK1 expression is elevated in OS

**Fig. 1:**
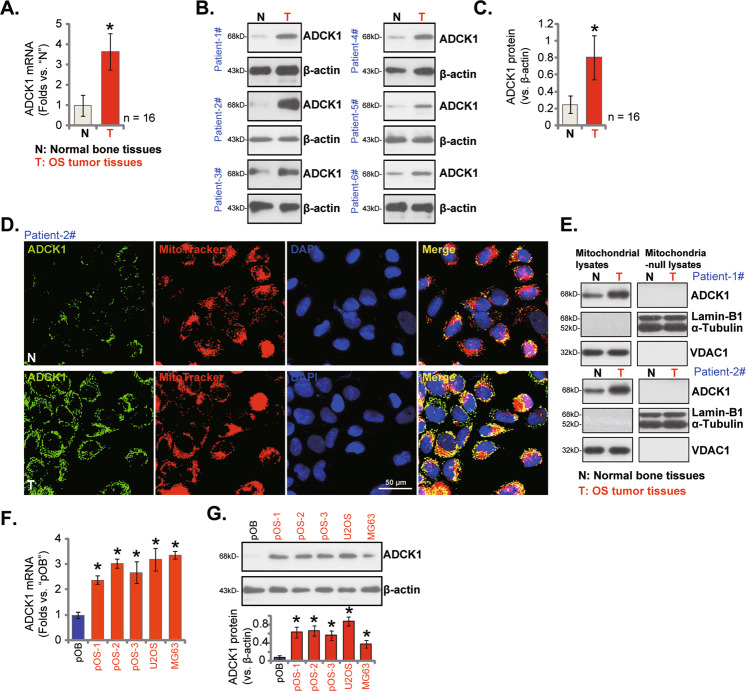
ADCK1 expression is elevated in OS. *ADCK1* mRNA and protein expression in OS tumor tissues (“T”) and matched adjacent normal bone tissues (“N”) from a total of 16 different primary OS patients (*n* = 16) was tested by qRT-PCR and Western blotting assays, and results quantified (**A**–**C**). The representative fluorescence images of ADCK1 protein (in green fluorescence), the MitoTracker Red (staining mitochondria, in red fluorescence) and DAPI (nuclei, in blue fluorescence) in the OS tumor slide (“T”) and the adjacent normal bone tissue slide (“N”) of Patient-2# were shown (**D**). Expression of the listed proteins in the mitochondrial lysates and the mitochondria-null lysates of tissues from two representative OS patients (Patient 1# and Patient 2#) was shown (**E**). *ADCK1* mRNA and protein expression in primary OS cells (pOS-1, pOS-2 and pOS-3), established OS cell lines (U2OS and MG63) and primary human osteoblasts (“pOB”) were tested (**F**, **G**). Data were presented as mean ± standard deviation (SD). *P < 0.05 vs. “N” tissues or “pOB” cells. Scale bar = 50 μm (**D**).

First we tested ADCK1 expression in human OS tissues. OS tumor tissues and matched adjacent normal bone tissues from a total of 16 different primary OS patients were obtained. The qRT-PCR assays were carried out to examine *ADCK1* mRNA expression. As shown *ADCK1* mRNA levels in OS tumor tissues (“T”) were significantly higher than those in the adjacent normal bone tissues (“N”) (Fig. 1A). Furthermore, ADCK1 protein levels were elevated in six representative OS patients, from Patient-1# to Patient-6# (Fig. 1B). We also combined the ADCK1 blotting data of all 16 pairs of tissue specimens and found that ADCK1 protein expression in OS tumor tissues was significantly higher than that in normal bone tissues (Fig. 1C).

Human tissue immuno-fluorescence results showed that ADCK1 protein (green fluorescence) co-localized with the MitoTracker Red (in red fluorescence) in OS tissue slides (Patient-2#) and matched normal bone tissue slides (Fig. 1D). The ADCK1 green fluorescence intensity in OS tumor slides was significantly higher than that in the normal bone tissue slides, further showing mitochondrial ADCK1 upregulation in the OS tissues (Fig. 1D).

The mitochondrial fraction lysates from fresh OS tumor tissues and normal bone tissues from Patient-1#/2# were obtained. As shown, ADCK1 protein was only enriched in the mitochondria lysates (Fig. 1E), indicated by the expression of VDAC1. Both Lamin-B1, the nuclear marker protein and α-Tubulin, the cytosol marker protein, were not present in the mitochondria lysates (Fig. 1E). Significantly, ADCK1 protein in the mitochondrial lysates of OS tumor tissues was again upregulated (Fig. 1E). ADCK1 protein was not detected in the mitochondria-null lysates of the tested human tissues, where α-Tubulin plus Lamin-B1 were present (Fig. 1E).

ADCK1 expression in different OS cells was tested as well. The primary human OS cells that were derived from three primary OS patients (pOS-1, pOS-2 and pOS-3 [17, 18]) as well as the established OS cell lines (U2OS and MG63) were tested. As shown *ADCK1* mRNA expression in the OS cells was significantly higher than that in the primary human osteoblasts (“pOB”) (Fig. 1F). Moreover, ADCK1 protein upregulation was detected as well in the primary and established OS cells (Fig. 1G). Thus, ADCK1 is upregulated in OS tissues and cells.

### ADCK1 silencing or KO produces significant anti-cancer activity in cultured OS cells

**Fig. 2:**
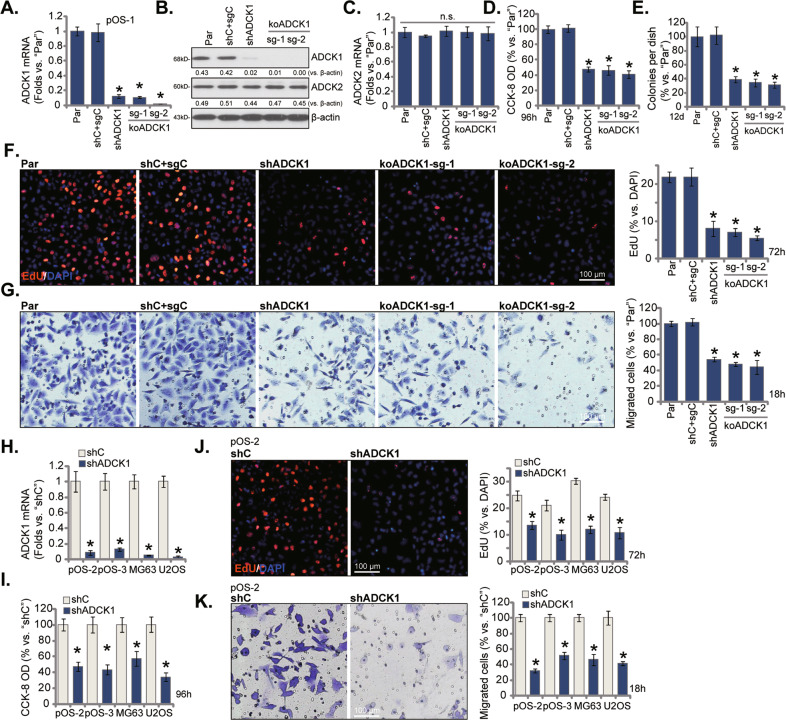
ADCK1 silencing or KO produces significant anti-cancer activity in cultured OS cells. Stable pOS-1 primary cells, bearing the lentiviral ADCK1 shRNA (“shADCK1”), the lentiviral CRISPR/Cas9 construct with sgRNA targeting *ADCK1*, “koADCK1-sg-1” and “koADCK1-sg-2”, or the lentiviral scramble control shRNA plus the CRISPR/Cas9 empty vector (“shC+sgC”), were established, mRNA and protein expression of ADCK1 and ADCK2 was tested by qRT-PCR and Western blotting assays (**A**–**C**); Cells were further cultured for applied time periods, colony formation (**D**), cell viability (CCK-8 OD, **E**), proliferation (nuclear EdU staining, **F**), migration (“Transwell” assays, **G**) were tested by the listed assays. The primary human OS cells (pOS-2 and pOS-3) as well as the established lines (MG63 and U2OS), expressing the lentiviral ADCK1 shRNA (“shADCK1”) or the lentiviral scramble control shRNA (“shC”), were established, *ADCK1* mRNA expression was tested by qRT-PCR assays (**H**); Cells were further cultured for applied time periods, cell viability (**I**), proliferation (**J**), and migration (**K**) were tested similarly. “Par” stands for the parental control cells. Data were presented as mean ± standard deviation (SD, *n* = 5). *P < 0.05 vs. “Par”/“shC” cells. “n.s.” stands for non-statistical difference (**C**). The experiments were repeated five times with similar results obtained. Scale bar = 100 μm (**F**, **G**, **J**, **K**).

Experiments were performed to test the potential function of ADCK1 in OS cells. To the primary human OS cells (pOS-1), the lentiviral particles containing the shRNA targeting *ADCK1* were added. Stable cells were established after puromycin selection, namely “shADCK1” cells. Alternatively, the lentiviral CRISPR/Cas9 construct encoding sgRNAs against *ADCK1*, sg-1, and sg-2 (two different sequences), were individually transduced to pOS-1 cells. Following ADCK1 KO screening, the single stable cells were obtained, namely “koADCK1” cells. The control pOS-1 cells were with the lentiviral scramble control shRNA plus the CRISPR/Cas9 empty vector (“shC+sgC”). *ADCK1* mRNA expression in stable cells was tested by the qRT-PCR assays and results showed that *ADCK1* mRNA reduced over 90% in the shADCK1 pOS-1 cells and the koADCK1 pOS-1 cells (Fig. 2A). ADCK1 protein expression, tested by Western blotting analyses, was dramatically downregulated as well (Fig. 2B). *ADCK2* and *ADCK3* mRNA (Fig. 2C) and protein (Fig. 2B) expression was not significantly altered by ADCK1 shRNA or CRISPR/Cas9 KO.

The number of viable pOS-1 cells, tested by the CCK-8 optical density (OD), was dramatically reduced after ADCK1 silencing or KO (Fig. 2D). The number of viable colonies was significantly decreased in shADCK1 and koADCK1 pOS-1 cells (Fig. 2E). The EdU-positive nuclei ratio reduction implied that ADCK1 depletion potently inhibited pOS-1 cell proliferation (Fig. 2F). Furthermore, ADCK1 silencing or KO potently suppressed pOS-1 cell in vitro migration (Fig. 2G). As shown, shC + sgC treatment did not significantly alter ADCK1-ADCK2 expression (Fig. 2A–C) and pOS-1 cell behaviors (Fig. 2D–G). In pOS-1 cells, shRNA-induced stable silencing of ADCK2 or ADCK3 (see the protein expression data in Fig. S1A) failed to significantly inhibit cell proliferation (Fig. S1B) and migration (Fig. S1C).

The potential role of ADCK1 silencing on other OS cells was studied next. The primary human OS cells that were derived from two other patients (pOS-2 and pOS-3 [17, 18]) as well as the established lines (MG63 and U2OS) were transduced with ADCK1 shRNA lentiviral particles, and stable cells established after puromycin selection (“shADCK1”). As compared to control OS cells with lentiviral scramble control shRNA (“shC”), *ADCK1* mRNA levels were dramatically decreased in shADCK1 OS cells (Fig. 2H). In the OS cells ADCK1 silencing largely inhibited cell viability (Fig. 2I), proliferation (Fig. 2J) and migration (Fig. 2K). Therefore, ADCK1 shRNA or KO resulted in potent anti-cancer activity in cultured OS cells.

### ADCK1 silencing or KO provokes caspase and apoptosis activation in OS cells

**Fig. 3:**
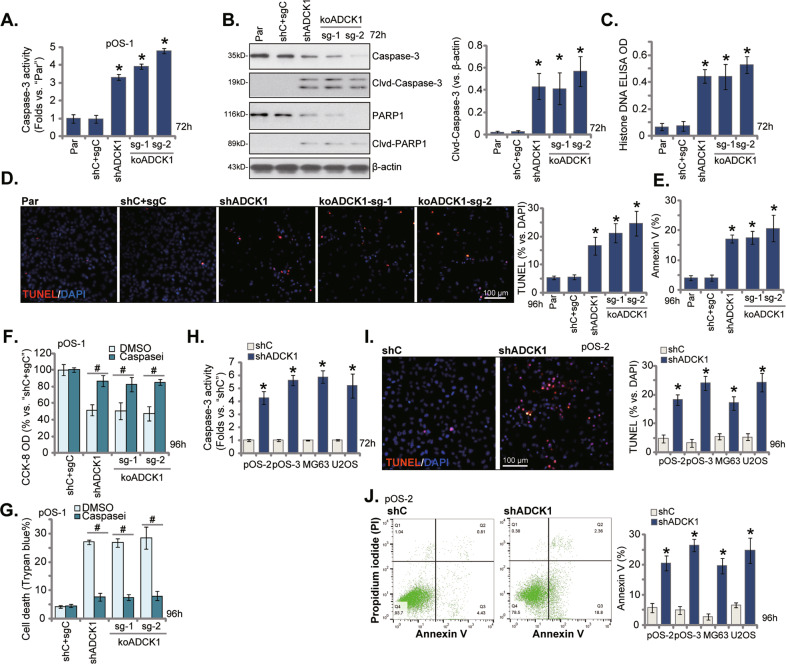
ADCK1 silencing or KO provokes caspase and apoptosis activation in OS cells. Stable pOS-1 primary cells, bearing the lentiviral ADCK1 shRNA (“shADCK1”), the lentiviral CRISPR/Cas9 construct with sgRNA targeting *ADCK1*, “koADCK1-sg-1” and “koADCK1-sg-2”, or the lentiviral scramble control shRNA plus the CRISPR/Cas9 empty vector (“shC+sgC”), were established and cultured for applied time periods; The relative caspase-3 activity (**A**), expression of apoptosis-associated proteins (**B**) and Histone-bound DNA contents (ELISA OD, **C**) were tested; Cell apoptosis was tested by nuclear TUNEL staining (**D**) and Annexin V FACS (**E**) assays. The above cells were treated with the pan caspase inhibitor z-VAD-fmk (“Caspasei, 40 μM) or the vehicle control (“DMSO”, 0.1%), and cultured for 96 h, cell viability and cell death were tested by CCK-8 (**F**) and Trypan blue staining (**G**) assays, respectively. The primary human OS cells (pOS-2 and pOS-3) as well as the established lines (MG63 and U2OS), expressing the lentiviral ADCK1 shRNA (“shADCK1”) or the lentiviral scramble control shRNA (“shC”), were established and cultured for applied time periods; The relative caspase-3 activity (**H**) and cell apoptosis (**I**, **J**) were tested similarly. “Par” stands for the parental control cells. Data were presented as mean ± standard deviation (SD, *n* = 5). *P < 0.05 vs. “Par”/“shC” cells. ^#^*P* < 0.05 vs. “DMSO” treatment (**F**, **G**). The experiments were repeated five times with similar results obtained. Scale bar = 100 μm (**D**, **I**).

ADCK1 silencing or KO resulted in potent anti-cancer activity in cultured OS cells. We next tested whether apoptosis was induced. As demonstrated the caspae-3 activity was significantly increased following ADCK1 silencing or KO in pOS-1 cells (Fig. 3A). Furthermore, levels of cleaved-caspasse-3, and cleaved-PARP1 were significantly augmented in shADCK1 pOS-1 cells and koADCK1 pOS-1 cells (Fig. 3B), where histone-bound DNA contents (ELISA assays) were elevated (Fig. 3C). Confirming apoptosis activation, we showed that TUNEL-positive nuclei ratio was significantly increased in shADCK1 and koADCK1 pOS-1 cells (Fig. 3D). Moreover, Fig. 3E demonstrated that ADCK1 silencing or KO increased the number of Annexin V-positive pOS-1 cells. These results confirmed that ADCK1 silencing or KO provoked caspase-apoptosis activation in pOS-1 cells. Unsurprisingly, shC + sgC treatment failed to induce significant apoptosis activation in pOS-1 cells (Fig. 3A–E).

Co-treatment with the pan caspase inhibitor z-VAD-fmk (“Caspasei”) potently ameliorated shADCK1- and koADCK1-induced viability (CCK-8 OD) reduction (Fig. 3F) and cell death (by recording Trypan blue-positive cells, Fig. 3G) in pOS-1 cells. These results implied that apoptosis activation should be the main mechanism responsible for ADCK1 depletion-induced cytotoxicity in OS cells.

In the shADCK1 pOS-1 cells, re-expression of a shRNA-resistant ADCK1 construct (“shR-ADCK1”) restored ADCK1 protein expression (Fig. S1D). Importantly, shADCK1-induced proliferation (nuclear EdU ratio) inhibition (Fig. S1E), migration reduction (Fig. S1F) and apoptosis (Fig. S1G) were reversed by shR-ADCK1 in pOS-1 cells.

In pOS-2 and pOS-3 primary cells as well as established cell lines (MG63 and U2OS), lentiviral shRNA-induced silencing of ADCK1 (“shADCK1”, see Fig. 2) increased caspase-3 activity (Fig. 3H) and induced apoptosis activation (Fig. 3I, J). The latter was confirmed by results from nuclear TUNEL staining (Fig. 3I) and Annexin V FACS (Fig. 3J) assays. These results together showed that ADCK1 silencing or KO provoked OS cell apoptosis.

### ADCK1 silencing or KO disrupts mitochondrial functions in OS cells

**Fig. 4:**
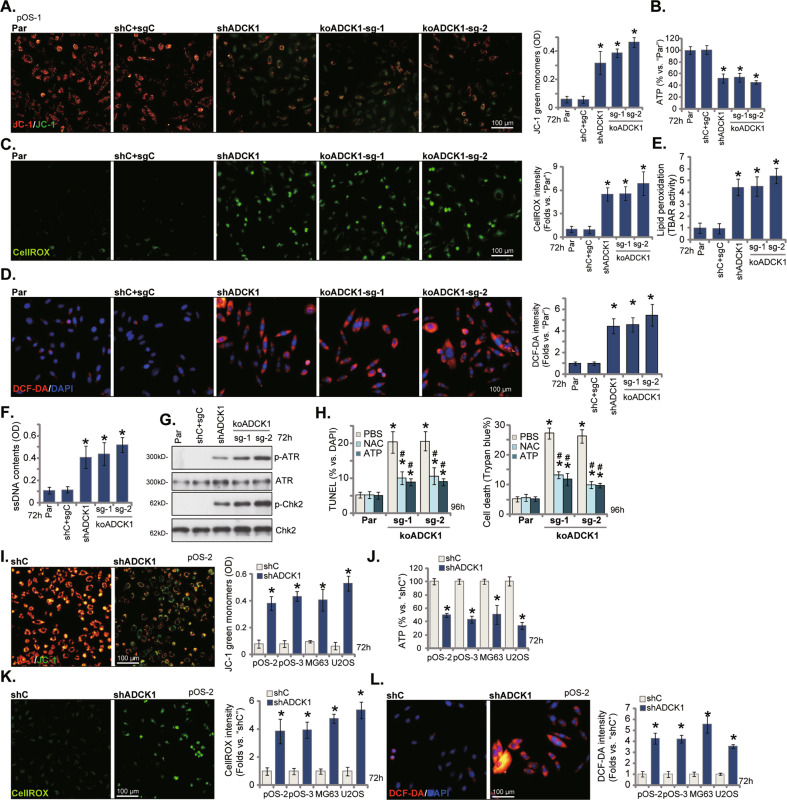
ADCK1 silencing or KO disrupts mitochondrial functions in OS cells. Stable pOS-1 primary cells, bearing the lentiviral ADCK1 shRNA (“shADCK1”), the lentiviral CRISPR/Cas9 construct with sgRNA targeting *ADCK1*, “koADCK1-sg-1” and “koADCK1-sg-2”, or the lentiviral scramble control shRNA plus the CRISPR/Cas9 empty vector (“shC+sgC”), were established and cultured for applied time periods; Mitochondrial depolarization (by measuring JC-1 green monomers intensity, **A**) and ATP contents (**B**) were tested; Oxidative injury was measured by CellROX staining (**C**) and DCF-DA staining (**D**) assays, with lipid peroxidation (TBAR activity assays, **E**) and single strand DNA (ssDNA) contents (ELISA assays, **F**) examined as well. Expression of the listed DNA damage proteins was shown (**G**). Stable pOS-1 primary cells, bearing the lentiviral CRISPR/Cas9 construct with sgRNA targeting *ADCK1*, “koADCK1-sg-1” and “koADCK1-sg-2”, were treated with the antioxidant N-Acetyl-L-cysteine (“NAC”, 500 μM), ATP (2 mM) or the vehicle control (“PBS”) and cultured for 96 h, cell death and apoptosis were tested by Trypan blue staining and TUNEL staining assays, respectively (**H**). The primary human OS cells (pOS-2 and pOS-3) as well as the established lines (MG63 and U2OS), stably expressing the lentiviral ADCK1 shRNA (“shADCK1”) or the lentiviral scramble control shRNA (“shC”), were established and cultured for applied time periods, mitochondrial depolarization (**I**), ATP contents (**J**) and ROS (CellROX intensity and DCF-DA intensity, **K** and **L**) were tested similarly. “Par” stands for the parental control cells. Data were presented as mean ± standard deviation (SD, *n* = 5). *P < 0.05 vs. “Par”/“shC” cells. ^#^*P* < 0.05 vs. “PBS” treatment (**H**). The experiments were repeated five times with similar results obtained. Scale bar = 100 μm (**A**, **C**, **D**, **J**–**L**).

ADCK1 is a protein essential for mitochondrial functions [14], we analyzed whether ADCK1 depletion could affect mitochondrial functions in OS cells. In pOS-1 primary cells, ADCK1 silencing or KO (Figs. 2 and 3) resulted in mitochondrial depolarization, which was evidenced by accumulation of JC-1 green monomers (Fig. 4A). Moreover, ATP contents were significantly decreased in shADCK1 and koADCK1 pOS-1 cells (Fig. 4B). CellROX staining assay results showed that ROS levels were significantly augmented in pOS-1 cells with ADCK1 silencing or KO (Fig. 4C). Further supporting oxidative injury, we found that DCFA-DA intensity was significantly intensified in shADCK1 and koADCK1 pOS-1 cells (Fig. 4D), which was accompanied by robust lipid peroxidation (TBAR activity increase, Fig. 4E). Accumulation of single strand DNA (ssDNA) indicated DNA breaks in pOS-1 cells with ADCK1 silencing or KO (Fig. 4F). Further supporting DNA damages, levels of phosphorylated- checkpoint kinase 2 (Chk2) and phosphorylated- ATM- and Rad3-Related (ATR) were significantly increased in the ADCK1-silenced/KO pOS1 cells (Fig. 4G). Total Chk2 and ATR expression was unchanged (Fig. 4G).

Importantly, shR-ADCK1 recovered mitochondrial functions and reversed mitochondrial depolarization (Fig. S1H) and ATP depletion (Fig. S1I) by ADCK1 silencing in pOS-1 cells. These results implied that ADCK1 shRNA or KO disrupted mitochondrial functions, causing mitochondrial depolarization, ATP depletion, ROS production, oxidative injury and DNA damage in pOS-1 cells.

In pOS-1 cells, co-treatment with the antioxidant NAC or exogenous ATP supplement ameliorated cell death (Trypan blue assays) and apoptosis (TUNEL staining assays) by ADCK1 KO through CRISPR/Cas9 (“sg-1/sg-2”) (Fig. 4H) and by shADCK1 (Fig. S2A). H_2_O_2_, serving as the positive control, induced robust cell death and apoptosis in pOS-1 cells (Fig. S2B). These results supported that ADCK1 depletion-induced mitochondrial dysfunction participated in subsequent OS cell death.

Similarly in pOS-2 and pOS-3 primary cells as well as in established OS cell lines (MG63 and U2OS), ADCK1 silencing induced mitochondrial depolarization (JC-1 green monomer accumulation, Fig. 4I), ATP depletion (Fig. 4J) and ROS production (CellROX/DCF-DA intensity increasing, Fig. 4K, L). shADCK1-induced cell death and apoptosis in pOS-2 cells were ameliorated by NAC and ATP as well (Fig. S2C).

### ADCK1 silencing sensitizes doxorubicin-induced apoptosis in primary OS cells

Doxorubicin (Dox), together with cisplatin and methotrexate, are still the standard and preferred chemotherapy for OS [25, 26]. It is estimated that over 40% of high-grade OS are unresponsive or only partially responsive to Dox [25, 26]. Dox is known to perturb mitochondrial structure and functions in OS cells [25–30]. Strategies targeting mitochondria have displayed synergistic activity in sensitizing Dox-induced OS cell apoptosis [25–31]. Considering that ADCK1 silencing disrupted mitochondrial functions (Fig. 4), we tested we tested whether it could induce Dox-dependent OS cell death.

**Fig. 5:**
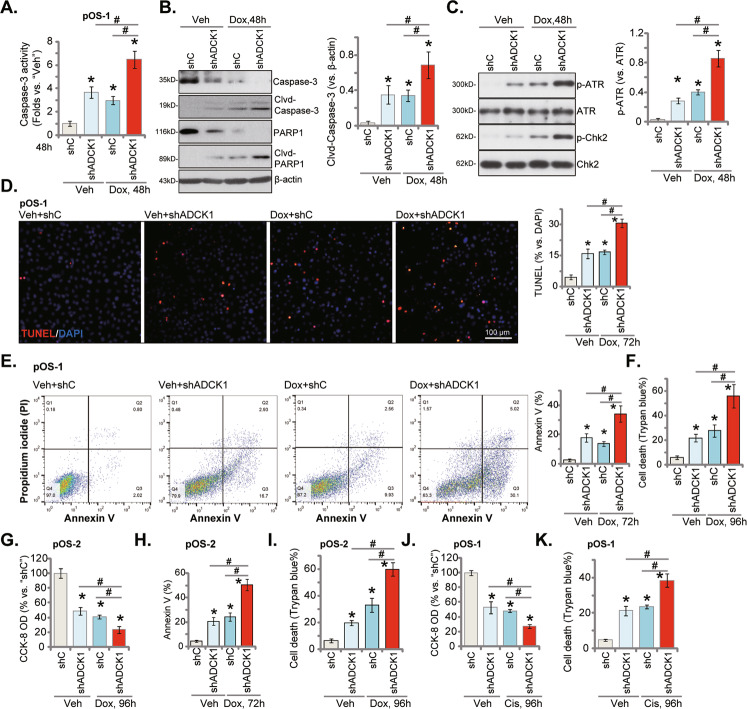
ADCK1 silencing sensitizes doxorubicin-induced apoptosis in primary OS cells. The pOS-1 (**A**–**F**) and pOS-2 (**G**–**I**) primary cells, expressing the lentiviral ADCK1 shRNA (“shADCK1”) or the lentiviral scramble control shRNA (“shC”), were treated with doxorubicin (Dox, 2 μg/mL) or vehicle control (“Veh”) for the designated hours, caspase-PARP1 activation was tested (**A**, **B**); Expression of listed DNA damage proteins was shown (**C**); Cell apoptosis and death were tested by the nuclear TUNEL staining/Annexin V FACS (**D**, **E**, **H**) and Trypan blue staining (**F**, **I**) assays, respectively, and cell viability examined by CCK-8 assays (**G**). The pOS-1 primary cells, expressing the lentiviral ADCK1 shRNA (“shADCK1”) or the lentiviral scramble control shRNA (“shC”), were treated with Cisplatin (Cis, 5 μg/mL) for 96 h, cell viability (**J**) and death (**K**) were tested similarly. *P < 0.05 vs. “Veh” treatment in “shC” cells. ^#^*P* < 0.05. Data were presented as mean ± standard deviation (SD, *n* = 5). Experiments were repeated five times with similar results obtained. Scale bar = 100 μm (**D**).

As demonstrated, doxorubicin-induced caspase-3 activation (Fig. 5A) as well as cleavages of caspase-3 and PARP1 (Fig. 5B) were augmented in ADCK1-silenced pOS-1 primary cells. Chk1 and ATR phosphorylation, indicating DNA damage intensity, was significantly increased following Dox+shADCK1 treatment, more potent than each single treatment (Fig. 5C). Chk1 and ATR expression was unchanged (Fig. 5C). Moreover, doxorubicin induced apoptosis activation in pOS-1 cells and increased the TUNEL-positive nuclei ratio (Fig. 5D) and Annexin V percentage (Fig. 5E). Importantly, ADCK1 silencing by shADCK1 intensified Dox-induced apoptosis (Fig. 5D, E). Moreover, Dox-induced pOS-1 cell death, tested by the increased Trypan blue staining, was potentiated by ADCK1 silencing as well (Fig. 5F). In pOS-2 primary cells, Dox-induced viability (CCK-8 OD) reduction (Fig. 5G), apoptosis (Annexin V staining increasing, Fig. 5H) and cell death (Trypan blue staining assays, Fig. 5I) were augmented by ADCK1 shRNA. Considering that shADCK1 by itself induced moderate but significant apoptosis activation, we concluded that ADCK1 could be an important resistance factor of Dox. Dox and ADCK1 shRNA can synergistically induce primary OS cell apoptosis. Interestingly, cisplatin-induced viability reduction (Fig. 5J) and cell death (Fig. 5K) were augmented by shADCK1 in pOS-1 cells as well.

### ADCK1 overexpression promotes OS cell proliferation and migration

**Fig. 6:**
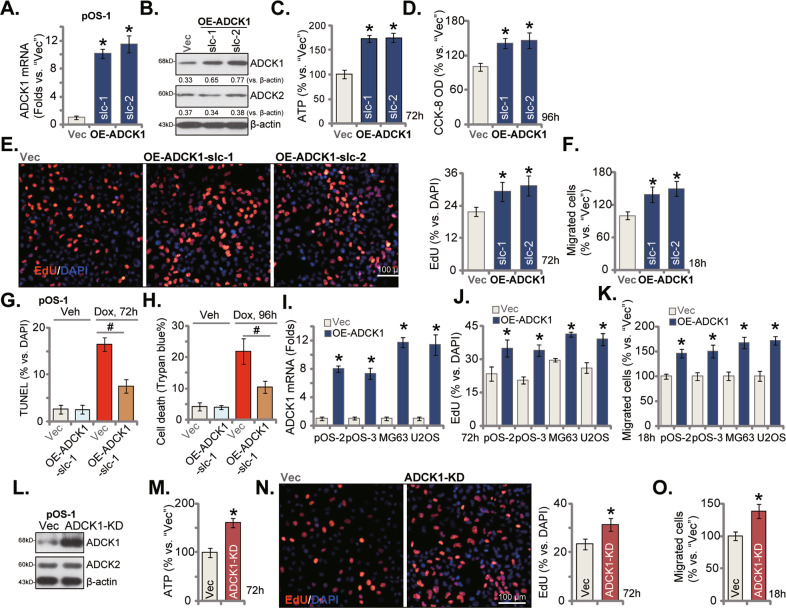
ADCK1 overexpression promotes OS cell proliferation and migration. The primary human OS cells (pOS-1, pOS-2 and pOS-3) as well as the established lines (MG63 and U2OS), bearing the ADCK1-expressing construct (“OE-ADCK1”, “slc-1/2” stands for two stable selections for pOS-1 cells) or the empty vector (“Vec”) were established, ADCK1 and ADCK2 expression was tested by qRT-PCR and Western blotting assays (**A**, **B**, **I**); Cells were further cultured for applied time periods, ATP contents (**C**), cell viability (CCK-8 OD, **D**), proliferation (by examining nuclear EdU staining, **E**, **J**) and migration (“Transwell” assays, **F**, **K**) were tested by the listed assays. OE-ADCK1-slc-1 pOS-1 cells or the Vec control cells were treated with doxorubicin (Dox, 2 μg/mL) or vehicle control (“Veh”) for the designated hours, cell apoptosis and death were tested by the nuclear TUNEL staining (**G**) and Trypan blue staining assays, respectively, and results were quantified (**H**). Stable pOS-1 cells, with the kinase dead mutant ADCK1 (“ADCK1-KD”, containing K183I, D315A, and D338N triple mutations) or the empty vector (“Vec”), were established, and expression of the listed protein was shown (**L**); Cells were further cultured for applied time periods, ATP contents (**M**), cell proliferation (by examining nuclear EdU staining, **N**), migrated cell number (“Transwell” assays, **O**) were tested similarly. Data were presented as mean ± standard deviation (SD, *n* = 5). *P < 0.05 vs. “Vec” cells. ^#^*P* < 0.05. The experiments were repeated five times with similar results obtained. Scale bar = 100 μm (**E**, **N**).

We proposed that ectopic overexpression of ADCK1 might exert opposite functions and should promote OS cell growth. Therefore, a lentiviral construct encoding the full-length *ADCK1* cDNA was transduced to pOS-1 cells. Two stable cell selections, OE-ADCK1-slc-1 and OE-ADCK1-slc-2, were established by puromycin. The qRT-PCR assays testing mRNA expression showed that *ADCK1* mRNA levels increased over 10 folds in the OE-ADCK1 pOS-1 cells (Fig. 6A). ADCK1 protein overexpression was detected as well in the OE-ADCK1 cells (Fig. 6B), where ADCK2 protein expression was unchanged (Fig. 6B). With ADCK1 overexpression the ATP contents were significantly increased in pOS-1 cells (Fig. 6C).

Functional studies showed that ectopic overexpression of ADCK1 in pOS-1 cells augmented cell viability (CCK-8 OD, Fig. 6D) and cell proliferation (EdU-positive nuclei increase, Fig. 6E). The quantified “Transwell” results further showed that ectopic overexpression of ADCK1 increased the number of migrated pOS-1 cells (Fig. 6F).

Significantly, Dox-induced apoptosis (TUNEL-positive nuclei increasing) (Fig. 6G) and death (Fig. 6H) were ameliorated in ADCK1-overexpressing pOS-1 cells (OE-ADCK1-slc-1). These results further supported that ADCK1 is a primary resistance factor of Dox.

The ADCK1-expressing lentiviral construct was also stably transduced to pOS-2 and pOS-3 primary cells as well as to the established cell lines (MG63 and U2OS), causing significant *ADCK1* mRNA upregulation (“OE-ADCK1”, Fig. 6I). ADCK1 overexpression promoted cell proliferation (EdU-positive nuclei increase, Fig. 6J) and migration (Fig. 6K) in the primary and established OS cells.

Interestingly, the pro-cancerous functions of ADCK1 appeared independent of the kinase activity of the protein. The kinase dead mutant ADCK1 (“ADCK1-KD”), containing triple mutations at the key amino acids related to the phosphotransferase activity of ADCK1 (K183I, D315A, and D338N) [14], was constructed and stably transduced to pOS-1 cells. Expression of ADCK1-KD was verified by Western blotting assays (Fig. 6L). ADCK1-KD, similar to overexpression of wild-type ADCK1, still increased ATP contents (Fig. 6M) and augmented pOS-1 cell proliferation (Fig. 6N) and migration (Fig. 6O).

### mTOR activation is important for ADCK1 expression in primary OS cells

**Fig. 7:**
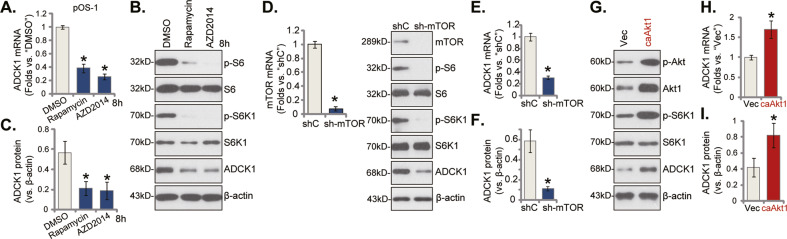
mTOR activation is important for ADCK1 expression in primary OS cells. The pOS-1 primary cells were treated with 100 nM of rapamycin or AZD2014 for 8 h, expression of *ADCK1* mRNA (**A**) and listed proteins (**B**, **C**) was shown. The pOS-1 primary cells, expressing the mTOR shRNA (“sh-mTOR”), the scramble control shRNA (“shC”) (**D**–**F**), the constitutively active Akt (“caAkt1”, S473D) or the empty vector (“Vec”) (**G**–**I**), were established; Expression of listed proteins and *ADCK1* mRNA was shown (**D**–**I**). Data were presented as mean ± standard deviation (SD, *n* = 5). “DMSO” stands for 1% DMSO. *P < 0.05 vs. “DMSO”/“shC”/“Vec” cells. The experiments were repeated five times with similar results obtained.

Activation of mTOR cascade is vital for OS tumorigenesis and progression [32–34]. mTOR hyperactivation is also required for sustained expression of several key oncogenic genes in OS [32–34]. We therefore analyzed whether mTOR activation is important for ADCK1 expression in OS cells. In pOS-1 primary cells treatment with the mTORC1 inhibitor rapamycin or the mTOR kinase inhibitor AZD2014 [35] potently deceased *ADCK1* mRNA expression (Fig. 7A). Phosphorylated S6 and S6K1 were blocked by rapamycin and AZD2014 (Fig. 7B). ADCK1 protein expression was significantly decreased after treatment with the mTOR inhibitors (Fig. 7B, C). Next, the mTOR shRNA lentiviral particles were transduced to pOS-1 primary cells, and stable cells established after puromycin selection (“sh-mTOR”). *mTOR* mRNA and protein expression as well as p-S6K1 and p-S6 levels were depleted in sh-mTOR pOS-1 cells (Fig. 7D). Importantly, *ADCK1* mRNA (Fig. 7E) and protein (Fig. 7D, F) expression was significantly downregulated in mTOR-silenced pOS-1 cells. These results further supported that mTOR activation is important for ADCK1 expression in OS cells.

Conversely, the constitutively active Akt (caAkt1, S473D [23]) adenovirus was transduced to pOS-1 primary cells, and the stable cells were established after puromycin selection. As compared to the vector control cells (“Vec”), Akt and S6K1 phosphorylation levels were significantly augmented in the caAkt1 pOS-1 cells (Fig. 7G). Notably, mTOR overactivation by caAkt1 significantly upregulated *ADCK1* mRNA (Fig. 7H) and protein (Fig. 7G, I) in pOS-1 cells. These results again supported that mTOR activation is important for ADCK1 expression in OS cells.

### ADCK1 inhibits OS xenograft growth in vivo

**Fig. 8:**
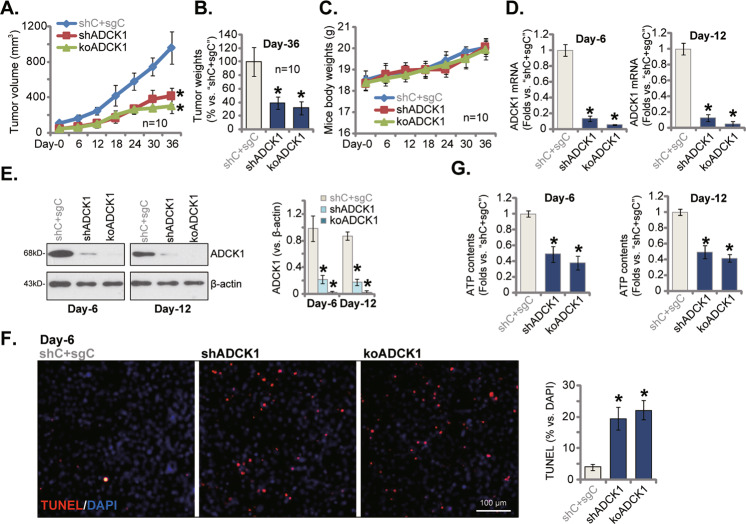
ADCK1 depletion inhibits OS xenograft growth in vivo. The primary pOS-1 cells bearing the ADCK1 shRNA (“shADCK1”), the lentiviral CRISPR/Cas9 construct with sgRNA (“sg-1”) targeting *ADCK1* (“koADCK1”) or the lentiviral scramble control shRNA plus the CRISPR/Cas9 empty vector (“shC+sgC”) were *s.c*. injected to the flanks of nude mice, and xenograft tumors established within two weeks (“Day-0”). Estimated tumor volumes (**A**) and mice body weights (**C**) were recorded every six days for 36 days in total; At “Day-36”, tumors of the three groups were isolated and weighted (**B**). At experimental “Day-6” and “Day-12”, one pOS-1 xenograft per group was isolated, and expression of listed genes and proteins in tumor lysates was shown (**D**, **E**), with ATP contents examined as well (**G**). The representative TUNEL-DAPI immuno-fluorescence xenograft slide images were presented as well (**F**), with TUNEL-positive nuclei ratio quantified (**F**). Data were presented as mean ± standard deviation (SD). *P < 0.05 vs. “shC+sgC” group. Scale bar = 100 μm (**F**).

At last, we tested the potential effect of ADCK1 on the growth of OS cells in vivo. The ADCK1 shRNA-expressing pOS-1 cells (“shADCK1”), the ADCK1 KO (with “sg-1”) pOS-1 cells (“koADCK1”) or the control cells with the lentiviral scramble control shRNA plus the CRISPR/Cas9 empty vector (“shC + sgC”) were *s.c*. injected to the flanks of nude mice (at 6 × 10^6^ cells per mouse). Within two weeks of cell injection, xenograft tumors were established and tumor recordings were initiated (labeled as “Day-0”). Tumor volumes were recorded every six days for 36 days in total (“Day-0” to “Day-36”). The tumor growth curve results were presented in Fig. 8A. Results showed that volumes of shADCK1 pOS-1 xenografts and koADCK1 pOS-1 xenografts were significantly lower than those of shC+sgC xenografts. Therefore, the growth of pOS-1 cells in vivo was largely inhibited after ADCK1 silencing or KO (Fig. 8A). At experimental “Day-36”, pOS-1 xenografts were removed carefully and weighted individually. As shown pOS-1 xenografts bearing ADCK1 shRNA or ADCK1 KO construct were significantly lighter than the control pOS-1 xenografts (Fig. 8B). The mice body weights, on the other hand, were not significantly different between the three groups (Fig. 8C).

To test the signaling changes, at experimental “Day-6” and “Day-12”, one pOS-1 xenograft per group was carefully isolated. The acquired six xenografts were cut into small pieces and dissolved into tissue lysis buffer. By employing qRT-PCR and Western blotting assays, tumor tissue lysates were analyzed. Results showed that *ADCK1* mRNA (Fig. 8D) and protein (Fig. 8E) levels were dramatically decreased in shADCK1 and koADCK1 pOS-1 xenografts. The tissue immunofluorescence images results demonstrated that TUNEL-positive staining was significantly increased in ADCK1-silenced or ADCK1-KO pOS-1 xenografts, further supporting apoptosis activation (Fig. 8F). In addition, ATP levels were reduced in pOS-1 xenografts bearing ADCK1 shRNA or KO construct (Fig. 8G). These findings in vivo were therefore in line with the in vitro signaling changes.

### ADCK1 KO inhibits pOS-1 xenograft in situ growth

**Fig. 9:**
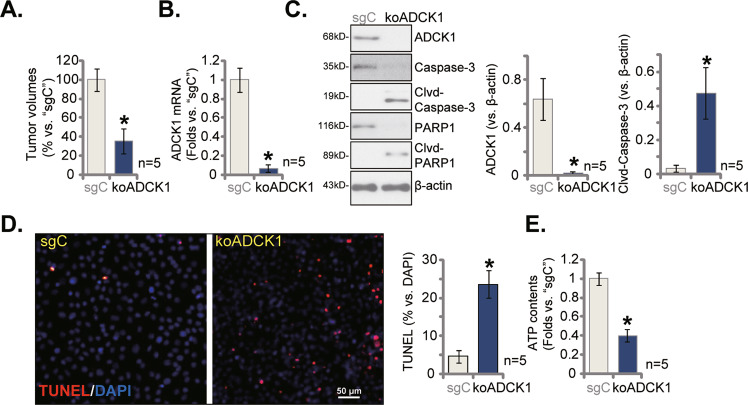
ADCK1 KO inhibits pOS-1 xenograft in situ growth. The sg-C expressing pOS-1 cells or the koADCK1 pOS-1 cells were injected to proximal tibia of the nude mice; After 30 days, in situ pOS-1 tumors were detected by X-ray, with its volumes recorded (**A**); Expression of listed genes in tumor lysates was shown (**B**, **C**), and ATP contents examined as well (**E**). The representative TUNEL-DAPI immuno-fluorescence images of the xenograft slides were presented (**D**), with TUNEL-positive nuclei ratio quantified (**D**). Data were presented as mean ± standard deviation (SD). *P < 0.05 vs. “sgC” group. Scale bar = 50 μm (**D**).

For in situ studies, the koADCK1 pOS-1 cells or the CRISPR/Cas9 empty vector-expressing pOS-1 cells (“sgC”) were injected to the proximal tibia of the nude mice (at 3 × 10^6^ cells per mouse). After 30 days, the in situ pOS-1 tumors were visualized under X-ray film). As shown the growth of the in situ pOS-1 tumors was hindered after ADCK1 KO (Fig. 9A). Tumor tissues were acquired and then tested by Western blotting and qRT-PCR analyses. We found that *ADCK1* mRNA (Fig. 9B) and protein (Fig. 9C) expression was depleted in the koADCK1 pOS-1 tumors, where cleaved-caspase-3 and cleaved-PARP1 levels were significantly increased (Fig. 9C). The TUNEL-positive nuclei percentage was significantly increased in koADCK1 tumors (Fig. 9D), further supporting apoptosis activation. ATP contents were again depleted in the ADCK1 KO in situ tumor tissues (Fig. 9E). These results showed that ADCK1 KO inhibited pOS-1 xenograft in situ growth.

## Discussion

OS is still the common primary bone tumor mainly detected in children, adolescents, and young adults [7, 36, 37]. In the past three decades 5-year overall survival for the pediatric OS has remained static and is close to 60–70% [7, 36, 37]. The vast majority of OS develop sporadically and are characterized by multiple gene rearrangements and a high degree of molecular heterogeneity [33, 37]. The results of this study provided a strong rationale for targeting ADCK1 in OS.

*ADCK1* mRNA and protein levels were significantly elevated in human OS tissues and multiple primary and established human OS cells (Fig. 1). Yet, low ADCK1 expression was detected in cancer-surrounding normal bone tissues and primary human osteoblasts (Fig. 1). In different OS cells, ADCK1 shRNA or KO potently inhibited colony formation, cell viability, proliferation and migration, while provoking caspase and apoptosis activation (Figs. 2 and 3). Conversely, stable and ectopic overexpression of ADCK1, using a lentiviral construct, exerted pro-cancerous activity in OS cells, promoting cancer cell proliferation and migration (Fig. 6). In vivo, the growth of subcutaneous pOS-1 xenografts in nude mice was largely inhibited after ADCK1 depletion using shRNA and CRISPR/Cas9 gene-editing methods (Fig. 8). Moreover, ADCK1 KO potently inhibited pOS-1 xenograft in situ growth in nude mice (Fig. 9). Therefore, these results highlighted ADCK1 as a novel and promising therapeutic target of OS.

One important finding of the present study is that overexpressed ADCK1 is a primary resistance factor of Dox in OS cells. In primary human OS cells, Dox-induced cell death and apoptosis were induced by shRNA-mediated silencing of ADCK1 (Fig. 5), but ameliorated after ADCK1 overexpression (Fig. 6). Further studies will be needed to explore the underlying mechanisms responsible for ADCK1-mediated Dox resistance in OS cells.

Due to mutation, depletion or amplification of various genes, sustained activation of mTOR signaling cascade positively contributed to OS progression [33, 38]. Studies have shown that mTOR-S6K1 cascade is activated in human OS, correlating with surgical stage, metastasis pattern, prognosis and overall survival of OS patients [39]. Conversely, mTOR inhibition with the selective inhibitors has demonstrated the promising potential in OS therapeutic research [39]. Yao et al., have shown that perifosine blocked Akt-mTOR complex 1 (mTORC1) signaling, promoting OS cell apoptosis and growth arrest [40]. XL388, a novel mTORC1/2 dual inhibitor, displayed therapeutic value in preclinical OS models, as it induced cytotoxic, cytostatic and pro-apoptotic activity in OS cells [41]. Drugs concurrently blocking PI3K and mTOR were able to induce apoptosis in primary human and murine OS cells [33].

Dysregulation and oncogenic activation of mTOR cascade is vital for the development and progression of OS [11, 33, 34], possibly due to its ability to enhance mRNA translation, ribosome biogenesis and protein stability of numerous oncogenic genes [11, 33, 34]. Indeed, a significant number of OS patients presented with genetically altered genes in the mTOR cascade [42]. Activation of mTOR also promotes lipogenesis, and energy metabolism to support OS growth [34, 43].

In the present study, we showed that mTOR activation is essential for ADCK1 expression in OS cells. The mTOR inhibitors, rapamycin and AZD2014, downregulated *ADCK1* mRNA and protein expression in primary OS cells (Fig. 7). Moreover, shRNA-induced silencing of mTOR led to ADCK1 reduction in primary OS cells (Fig. 7). Conversely, elevated *ADCK1* mRNA and protein expression was detected in caAkt1-expressing pOS-1 cells with mTOR overactivation (Fig. 7). The underlying signaling mechanisms of mTOR-dependent ADCK1 expression in OS cells certainly require further throughout studies.

ADCK1 is a mitochondrial protein kinase with its functions largely unknown. It has been shown that *Drosophila* ADCK1 mutants died as second instar larvae with molting defects and tracheal breaks [13], which was correlated with reduced signaling of the steroid hormone ecdysone [13]. RNAi studies indicated that ADCK1 was both necessary and sufficient for viability in *Drosophila* [13]. ADCK1 is essential in maintaining the cristae structure of mitochondria and is involved in mitochondrial fusion/fission [14]. ADCK1 silencing increased fusion, causing ATP depletion, ROS generation, whereas ADCK1 over-expression increased mitochondrial fission [14].

In the eukaryotic cells mitochondrion is the principal location for ATP production and macromolecules biosynthesis. Recent studies have demonstrated that increased mitochondrial bioenergetics, dynamics, and signaling are essential for OS tumorigenesis [44–46]. Here we found that ADCK1 is important for maintaining of the mitochondrial functions in OS cells. ADCK1 shRNA or KO induced mitochondrial dysfunctions in OS cells, causing mitochondrial membrane potential reduction, ATP depletion, ROS production and oxidative injury (Fig. 4). ATP depletion was also detected in ADCK1-depleted OS xenograft tissues (Figs. 8 and 9). Conversely, ectopic overexpression of ADCK1 increased ATP contents in OS cells (Fig. 6). Therefore maintaining mitochondrial function could be the key mechanism responsible for ADCK1-driven OS cell growth. It is also possible that other mechanisms could also be involved in ADCK1-driven OS progression, and further studies are needed.

## Conclusion

ADCK1 is an important mitochondrial protein required for OS cell growth and represents a novel and promising therapeutic target of OS.

## Supplementary information

Supplementary Figures including original data

Author contribution form

aj-checklist form

## Data Availability

All data are available upon request.
