# Supplementary material for: ADCK1 is a potential therapeutic target of osteosarcoma
Source: Cell Death Dis. 2022 Nov 12;13(11):954. doi: 10.1038/s41419-022-05401-8 (PMC9653483; doi:10.1038/s41419-022-05401-8)

Figure S1.

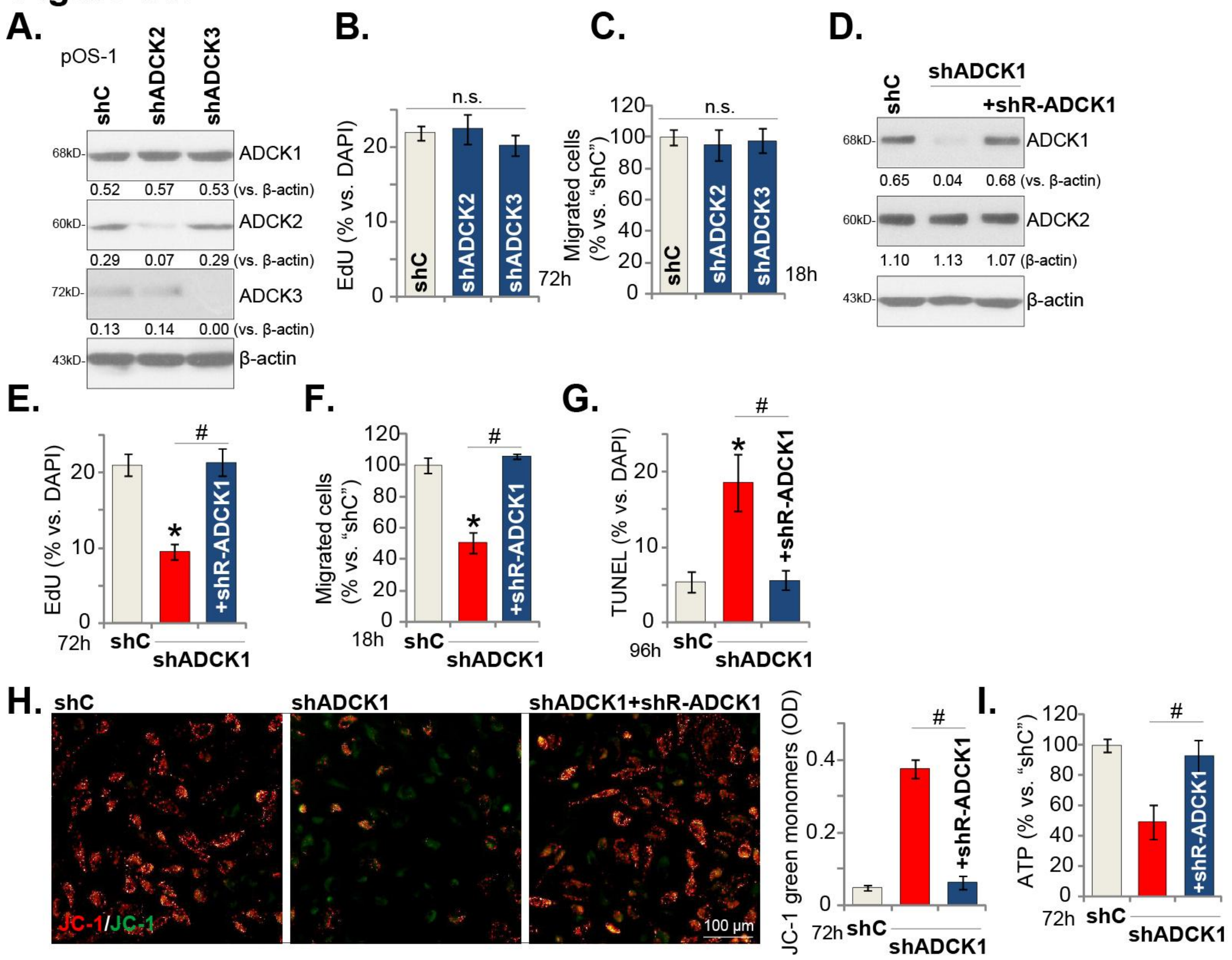

Figure S1. The pOS-1 primary human OS cells, expressing the lentiviral ADCK2 shRNA ("shADCK2"), the lentiviral ADCK3 shRNA ("shADCK3") or the lentiviral scramble control shRNA ("shC"), were established; Expression of listed proteins was shown (**A**); Cells were further cultured for applied time periods, cell proliferation and migration were examined by the nuclear EdU staining (**B**) and "Transwell" (**C**) assays, respectively, and results were quantified. The pOS-1 primary cells expressing the lentiviral ADCK1 shRNA ("shADCK1") were further infected with or without the lentivirus encoding the shRNA-resistant ADCK1-expressing construct ("shR-ADCK1"), and puromycin added to select stable cells; Expression of listed proteins was shown (**D**); Cells were further cultured for applied time periods, cell proliferation, migration and apoptosis were examined the nuclear EdU staining (**E**), "Transwell" (**F**) and the nuclear TUNEL staining (**G**) assays, respectively, and results were quantified. Mitochondrial depolarization (by measuring JC-1 green monomers intensity, **H**) and ATP contents (**I**) were

tested as well; “n.s.” stands for non-statistical difference (**B** and **C**). * ***P*** < 0.05 vs. “shC” cells (**E-I**). # ***P*** < 0.05. Data were presented as mean ± standard deviation (SD, n=5). The experiments were repeated five times with similar results obtained. Scale bar = 100 μm.

Figure S2

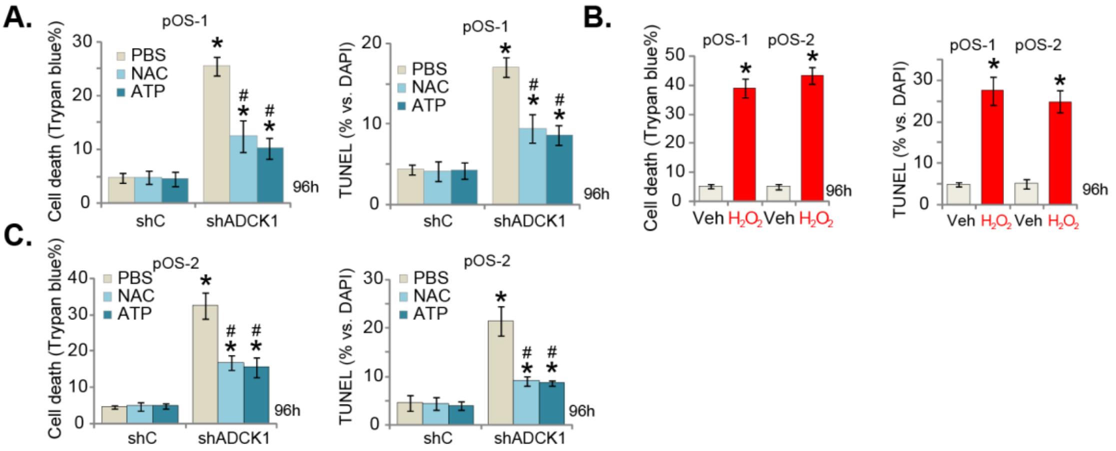

Figure S2. pOS-1 cells (**A**) or pOS-2 cells (**C**) with shADCK1 or control shRNA ("shC") were treated with the antioxidant N-Acetyl-L-cysteine ("NAC", 500 μM), ATP (2 mM) or PBS, and cultured for 96h, cell death and apoptosis were tested by Trypan blue staining and TUNEL staining assays, respectively. pOS-1 cells or pOS-2 cells were treated with hydrogen peroxide (H_2O_2, 400 μM) or vehicle ("Veh") and cultivated 96h, and cell death and apoptosis were tested similarly (**B**). * $P < 0.05$ vs. "shC" cells/"Veh". # $P < 0.05$ vs. "PBS" treatment (**A** and **C**). The experiments were repeated five times with similar results obtained.

Figure S3: The un-cropped blotting images of the present study.

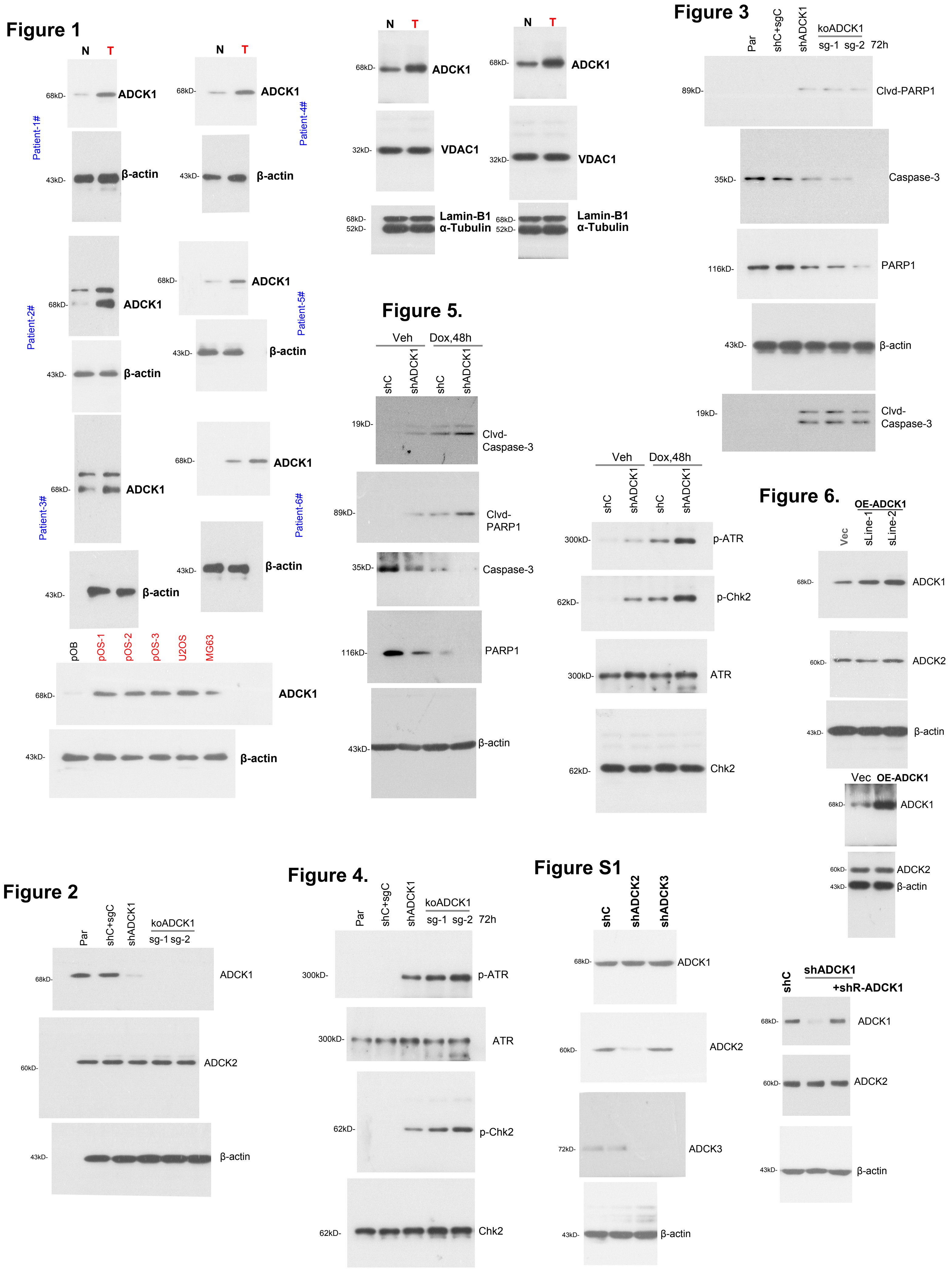

Figure 7

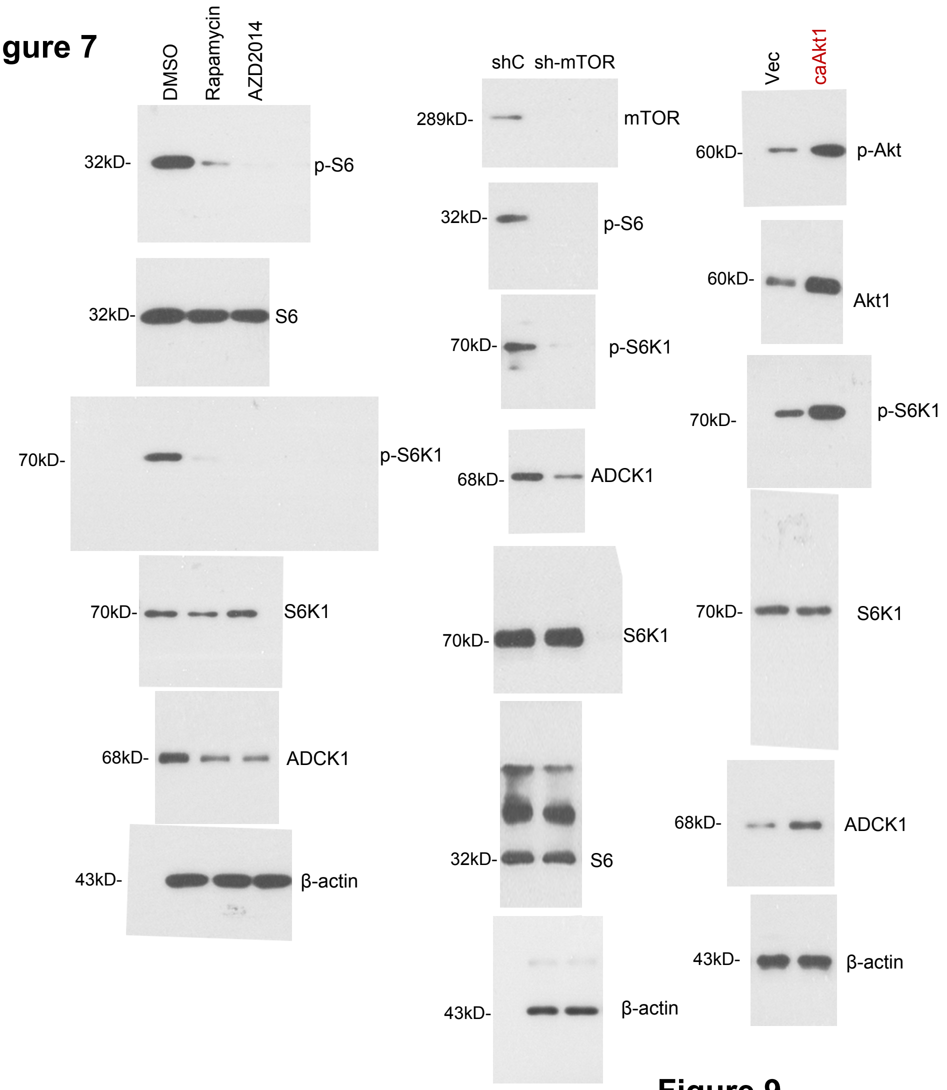

Figure 8

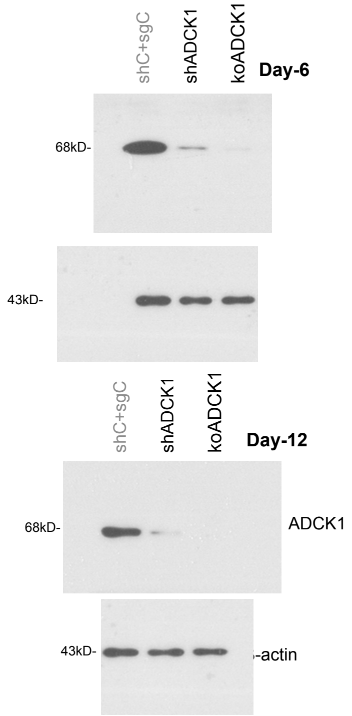

Figure 9

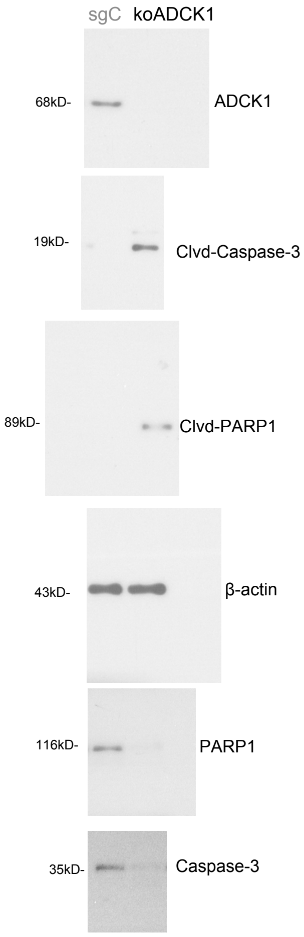

Supplement: Supplementary file 1 — Supplementary Figures including original data [file 41419_2022_5401_MOESM1_ESM.pdf]
