# Supplementary material for: ADCK1 is a potential therapeutic target of osteosarcoma
Source: Cell Death Dis. 2022 Nov 12;13(11):954. doi: 10.1038/s41419-022-05401-8 (PMC9653483; doi:10.1038/s41419-022-05401-8)
Supplement: Supplementary file 2 — Author contribution form [file 41419_2022_5401_MOESM2_ESM.pdf]

DECLARATION OF CONTRIBUTIONS TO ARTICLE

ADMC

Manuscript Number:	Journal Name:	
	Cell Death & Disease	(the 'Journal')

Proposed Title of the Contribution:

ADCK1 is a potential therapeutic target of osteosarcoma (the 'Contribution)

Author(s):

Bao-biao Zhuo, Lun-qing Zhu, Chen Yao, Xi-hua Wang, Shi-xian Li, Rong Wang, Yuan Li and Zhuo-yan Ling (the 'Authors')

For all *CDDis* articles, each person named as an author in the published version must be able to show he or she has contributed substantially to the article.

Authorship credit should be based on 1) substantial contributions to conception and design, acquisition of data, or analysis and interpretation of data; 2) drafting the article or revising it critically for important intellectual content; and 3) final approval of the version to be published. Authors should meet conditions 1, 2 and 3.

Any person who cannot be shown to have made a substantial contribution to the article cannot be listed as an author in the final version. The name of any person who is deemed to have made a minor contribution can, however, appear in the Acknowledgments section of the article.

Please complete the table below to indicate the contributions of all named authors to the manuscript.

Author Full Name:	Specification of Contribution to the Manuscript:
Bao-biao Zhuo	Functional assays in OS and normal cells, establishing OS xenografts, testing signalings and cell death/apoptosis, drafting the article and revising it, with final approval of the version submitted to the journal.
Lun-qing Zhu	Functional assays in OS and normal cells, testing signalings and cell death/apoptosis, drafting the article and revising it, with final approval of the version submitted to the journal.
Chen Yao	Preparing and verifying the virus, sequences, constructs, and primers, drafting the article and revising it, with final approval of the version submitted to the journal.
Xi-hua Wang	Establishing OS xenografts, testing signaling in OS cancer tissues, drafting the article and revising it, with final approval of the version submitted to the journal.
Shi-xian Li	Establishing OS xenografts, Figure organization, involved in drafting the article and revising it critically for important intellectual content, and with final approval of the version submitted to the journal.
Rong Wang	Study conception and design, and data analysis, Figure organization, involved in drafting the article and revising it critically for important intellectual content, and with final approval of the version submitted to the journal.
Yuan Li	Study conception and design, and data analysis, Figure organization, involved in drafting the article and revising it critically for important intellectual content, and with final approval of the version submitted to the journal.
Zhuo-yan Ling	Study conception and design, and data analysis, Figure organization, involved in drafting the article and revising it critically for important intellectual content, and with final approval of the version submitted to the journal.

Please complete the table below to indicate the contributions of all named authors to the figures.

Figure 1:

Bao-biao Zhuo, Lun-qing Zhu, Chen Yao, Xi-hua Wang, Rong Wang, Yuan Li and Zhuo-yan Ling

Figure 2:

Bao-biao Zhuo, Lun-qing Zhu, Chen Yao, Xi-hua Wang, Shi-xian Li, Yuan Li and Zhuo-yan Ling

Figure 3:

Bao-biao Zhuo, Lun-qing Zhu, Chen Yao, Shi-xian Li, Rong Wang, Yuan Li and Zhuo-yan Ling

Figure 4:

Bao-biao Zhuo, Lun-qing Zhu, Chen Yao, Xi-hua Wang, Shi-xian Li, Rong Wang, Yuan Li and Zhuo-yan Ling

Figure 5:

Bao-biao Zhuo, Lun-qing Zhu, Shi-xian Li, Rong Wang, Yuan Li and Zhuo-yan Ling

Figure 6:

Bao-biao Zhuo, Lun-qing Zhu, Chen Yao, Rong Wang, Yuan Li and Zhuo-yan Ling

Figure 7-9

Bao-biao Zhuo, Lun-qing Zhu, Chen Yao, Xi-hua Wang, Shi-xian Li, Rong Wang, Yuan Li and Zhuo-yan Ling

Signed for and on behalf of the Author(s):	Print Name:	Date:
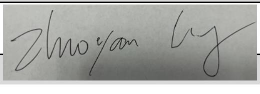	Zhuo-yan Ling	05-01-2022
